# Toward the development of a polarimetric tool to diagnose the fibrotic human ventricular myocardium

**DOI:** 10.1117/1.JBO.27.5.055001

**Published:** 2022-05-13

**Authors:** Twinkle Bagha, Arif Mohd. Kamal, Uttam M. Pal, Prasanna Simha Mohan Rao, Hardik J. Pandya

**Affiliations:** a Indian Institute of Science, Department of Electronic Systems Engineering, Bangalore, Karnataka, India; b Indian Institute of Information Technology Design and Manufacturing, Kancheepuram, Tamil Nadu, India; c Sri Jayadeva Institute of Cardiovascular Sciences and Research, Bangalore, Karnataka, India

**Keywords:** cardiac fibrosis, myocardial tissues, myxomatous valve, optical polarimetry, rheumatic heart disease

## Abstract

**Significance:**

Optical polarimetry is an emerging modality that effectively quantifies the bulk optical properties that correlate with the anisotropic structural properties of cardiac tissues. We demonstrate the application of a polarimetric tool for characterizing healthy and fibrotic human myocardial tissues efficiently with a high degree of accuracy.

**Aim:**

The study was aimed to characterize the myocardial tissues from the left ventricle and right ventricle of N=7 control and N=10 diseased subjects. The diseased subjects were composed of two groups: N=7 with rheumatic heart disease (RHD) and N=3 with myxomatous valve (MV) disease.

**Approach:**

A portable, affordable, and accurate linear polarization-based diagnostic tool is developed to measure the degree of linear polarization (DOLP) of the myocardial tissues while working at a wavelength of 850 nm.

**Results:**

The sensitivity, specificity, and accuracy of the polarimetric tool in distinguishing the control group from the RHD group were found to be 73.33%, 76.92%, and 75%, respectively, and from the MV group were 91.6%, 62.5%, and 80%, respectively, which demonstrates the efficacy of the polarimetric tool to distinguish the healthy myocardial tissues from diseased tissues.

**Conclusions:**

We have successfully developed a polarimetric tool that can aid cardiologists in characterizing the myocardial tissues in conjunction with endomyocardial biopsy. This work should be followed up with experiments on a large cohort of control and diseased subjects. We intend to create and develop a probe to quantify the DOLP of *in vivo* heart tissue during surgery.

## 1 Introduction

According to the Global Burden of Disease report, cardiovascular diseases (CVDs) are still the main cause of disease burden around the world.^1^ Total CVD prevalence approximately doubled from 271 million in the year 1990 to 523 million in the year 2019.^1^ Also, the number of fatalities due to CVDs has gradually scaled up from 12.1 million in the year 1990 to 18.6 million in the year 2019.^1^

Myocardial disorders are generally characterized by alterations in tissue compositions, such as the development of fibrosis, edema, or any infiltration with inflammatory cells, iron, fat, or amyloid.^2^ These alterations in the extracellular matrix (ECM) can cause heart systolic and/or diastolic dysfunction, increasing the risk of unfavorable cardiovascular events.^2^ As a result, early detection of structural cardiac abnormalities has major diagnostic and prognostic value.

Fibrosis is the production of fibrous connective tissues in response to an injury, which is characterized by the accretion of ECM components, mainly collagen, near the site of damage.^3^ Fibrosis is an essential component of tissue restoration and wound healing. When fibrosis advances uncontrollably, the damaged tissue becomes permanently stiffened, leading to organ failure and death.^4^ Fibrotic disorders kill about 800,000 people each year, accounting for 45% of fatalities globally.^4^ A significant number of fibrosis-related fatalities are caused by fibrosis of the heart and lungs.^3^

Cardiac fibrosis is a scarring process characterized by enhanced type-I collagen deposition, and activation of cardiac fibroblasts, followed by their differentiation into myofibroblasts, occurring in the cardiac muscle.^4^ Cardiomyocyte degradation and the formation of fibrosis are both important components of cardiac remodeling.^5^ Cardiac remodeling has been described by the alterations occurring at cellular and molecular levels that manifest clinically as modifications in the size, shape, and/or functioning of the heart following damage.^6^ These pathological alterations enhance the stiffness of the ECM, causing decreased heart function.^4^ Over time, the buildup of fibrotic proteins results in persistent tissue remodeling and significant organ damage.^7^ If this remodeling is irreversible, it can lead to organ dysfunction and, in extreme cases, death if left untreated.^7^ Hence, analyzing cardiac tissues at an early stage of the disease can be life-saving.

Endomyocardial biopsy is the gold standard for detecting myocardial fibrosis,^2^^,^^8^ in which a tiny ($1\;{\mathrm{mm}}^{3}$) sample is excised from the myocardial septum adjacent to the right ventricle (RV). Next, the specimen is examined using Masson trichome staining, followed by quantifying the volume fraction of collagen in tissue samples using quantitative morphometry and staining with picrosirius red.^8^ As an invasive method, endomyocardial biopsy offers limited accuracy due to sampling errors and is practically infeasible in daily clinical routine.^9^ Moreover, an endomyocardial biopsy is incapable of identifying fibrosis of the whole LV.^4^ Hence, there is an urgent need to develop a non-invasive tool for evaluating fibrosis *in-vivo*, which can aid in detecting fibrosis early and accurately.

**Fig. 1:**
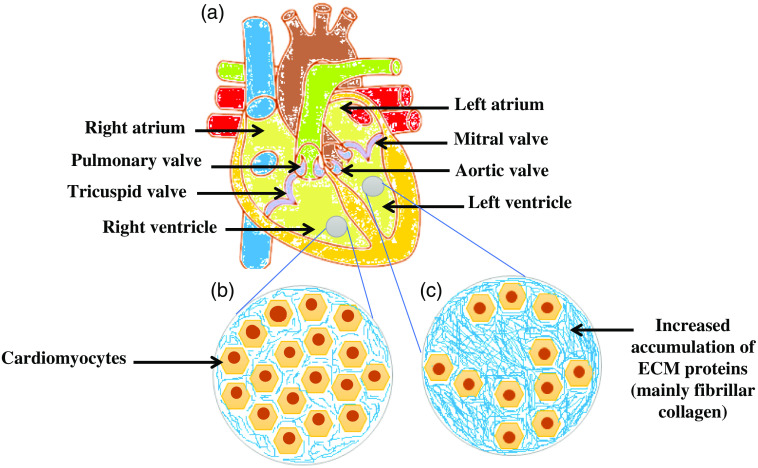
(a) Schematic of the human heart showing all four chambers and valves; (b) healthy cardiac tissue; and (c) diseased cardiac tissue considering RV as normal and LV as diseased.

Optical polarimetry, which assesses a sample’s effect on polarized light, has emerged as an effective approach for the structural characterization of cardiac tissues, quantifying the extent of the underlying fibrous structure. Because optical polarimetry is highly sensitive to anisotropy, numerous researchers have used it to characterize diverse biological tissues. The myocardium is made up of sheets of cardiac muscle fibers that wrap around the ventricles and atriums.^10^ Cardiac tissue is composed of fibrous components (such as cardiomyocytes and collagen fibers) that are highly anisotropic, which is significant for assessing cardiac disorders using optical polarimetry.^11^ The healthy zones of heart tissues are predominantly constituted of well-organized arrays of cardiomyocytes [Fig. 1(b)], resulting in higher linear birefringence values than the pathological zones, where severe disruption of the normally well-ordered architecture leads to a commensurate loss of cardiac tissue anisotropy.^11^ In diseased cardiac tissues [Fig. 1(c)], the rise in collagen concentration (i.e., fibrosis) is the most common and significant contribution of ECM proteins to CVD.^5^ Even in the absence of myocyte loss, an abnormal buildup of fibrillar collagen impairs heart function.^6^ In the interstitial and perivascular areas, the normal collagen volume percentage, as measured by morphometry, is about 4%.^5^ Depolarization directly correlates with the anisotropic structure of the constituents of heart tissue, which is considered a prominent metric in tissue optical polarimetry (e.g., myocardium).^11^

Bickel et al.^12^ demonstrated that polarization in light scattering could be used as an effective tool in biological study. Various biological tissues, including tissues from cardiac muscle, brain, liver, kidney cortex, loin muscle, and tendon of pigs,^13^^–^^16^ tissues from heart, brain, retina, kidney, and spleen of rats,^17^ cardiac tissues of Lewis rats,^18^^,^^19^ human breast cancer tissues,^20^^–^^22^ and invasive breast cancer specimens,^23^^–^^25^ have been characterized using a wide variety of optical modalities such as polarimetric light microscopy (PLM),^23^^–^^25^ linear polarization spectroscopy,^23^^–^^25^ Mueller polarimetry,^26^ near-infrared spectroscopy (NIRS),^20^^–^^22^^,^^27^ and optical coherence tomography (OCT).^13^^,^^14^

Sprenger et al.^23^ devised a quantitative approach for assessing the tumor’s stroma ratio by combining PLM and linear polarization in breast cancer specimens. The proposed polarimetric technique distinguishes between low and high stroma groups, aiding in the reduction of picture contrast changes caused by polarizer orientation in relation to birefringent structures in breast tissues, allowing for a high-contrast view.^23^ In human invasive breast cancer samples, Westreich et al.^24^ showed the use of linearly polarized light for imaging and analyzing morphological features related to stromal density and alignment. The advantages of PLM and linear polarization were also investigated by Jones et al.^25^ for quantifying signatures of tumor-stromal architecture to differentiate between sclerotic and myxoid breast cancer stroma. This method produced high contrast images of stromal morphology in breast tumor specimens.^25^

Sanchez-Cano et al.^17^ developed an optical fiber-based supercontinuum setup consisting of a spectrophotometer capable of measuring spectra ranging from 1100 to 2300 nm for characterizing the absorption, attenuation, and scattering spectral coefficients of *ex vivo* rat tissues of heart, kidney, brain, retina, and spleen. For detecting glucose in *ex vivo* human gingival tissues, John et al.^28^ used a wideband supercontinuum laser source filtered by bandpass filters with multiple wavelengths of 1300, 1580, and 2100 nm based on low coherence interferometry. Sharma et al.^26^ reported the use of Mueller polarimetry along with image analysis in bulk tissues as an alternative technology to tissue histology for surgically resected breast and colon tissues. It was asserted that polarimetric analysis of bulk tissue provides a comprehensive examination of various optical properties of tissues for diagnosis.^26^

Tissue polarimetry is an optical diagnostic approach that exploits the optical polarization’s sensitivity to detect early changes in the morphology of tissues.^29^ Also, the use of polarized light to probe the tissue has been proved to be a quick and effective way for non-invasive tissue diagnostics.^30^ Kamal et al.^22^ designed a small, inexpensive, and easy-to-use system (POLS-NIRDx) for performing polarization-sensitive NIRS to distinguish between the malignant and the adjacent normal formalin-fixed breast biopsy tissues by quantifying the degree of linear polarization (DOLP) and degree of circular polarization (DOCP), both at 850 and 940 nm.

Borovkova et al.^31^ investigated the application of wide-field Mueller imaging polarimetry by introducing a label-free polarimetric technique to quantify the degree of depolarization of amyloid-beta plaques deposits throughout the grey matter and hippocampi observed in Alzheimer’s disease. Lee et al.^32^ demonstrated the use of Mueller polarimetry imaging technique in the visible spectrum to determine the cervical ECM remodeling by performing non-contact collagen scoring of cervix tissues in a mice model during normal pregnancy, which is not detected in standard intensity images. The linear depolarization images of mice’s cervix tissues and total linear retardance were used as collagen scoring metrics.^32^

Ivanov et al.^33^ explored the applicability of polarized light to analyze the abnormalities in the *ex vivo* colon samples by combining two-dimensional (2D) Stokes vector polarimetry mapping and Poincaré sphere approaches and comparing the linear and circular polarization states of the samples. Later, Stokes–Mueller matrix polarimetry was combined with filtering and decomposition algorithms, demonstrating that polarization and depolarization metrics can be utilized as cancer biomarkers for differentiating cancerous from healthy colon tissues.^29^

Ushenko et al.^34^ developed a polarization-holographic Mueller matrix method for the layered mapping of the degree of depolarization of the diffuse layers of biological tissues, such as myocardial fibrillar and parenchymal liver tissues for assessing the three-dimensional (3D) morphology of biological tissues. Roa et al.^35^ proposed a methodology integrating Mueller matrix polarimetry with convolutional neural networks and K-nearest neighbor techniques to detect and classify collagen and elastin in mice cervix by developing a Mueller matrix micromesoscope system. In this study, total reflectance was quantified along with analyzing the accuracy of results by calculating the structural similarity index, peak signal-to-noise ratio, and mean square error of the images.^35^

Gonzalez et al.^36^ developed a low-cost snapshot Mueller matrix polarimeter for imaging the *ex vivo* pig cervix, which could be used for cervical screening in low-resource settings. The polarization properties were quantified using the Stokes vector, and the depolarization and retardance data were retrieved using Mueller matrix decomposition.^36^ Mueller matrix microscopy, as well as logarithmic decomposition and polarized Monte Carlo (MC) modeling, were proposed by Li et al.^30^ for qualitative and quantitative analysis of thin human skin tissues, for extracting the details about the tissue morphology, which is not provided by white-light microscopy.

Panigrahi and Gioux^37^ showed that machine-learning methods could replace nonlinear models in estimating the diffuse reflectance and optical properties such as absorption and reduced scattering from the diffuse reflectance images, combining spatial frequency domain imaging with an ensemble learning algorithm, i.e., the random forest regressor (RFR) method. Nguyen et al.^38^ used the diffuse reflectance spectroscopy (DRS) technique to extract the physiological parameters, such as melanin concentration, blood volume fraction, oxygen saturation, reduced scattering coefficient, and reduced scattering exponent using various machine-learning models for tissue diagnosis. Machine-learning models yield lower errors and provide faster runtime compared to the MC lookup table-based model for extracting the physiological parameters from the DRS data using six wavelengths.^38^

Alali^15^ optically examined isotropic pig tissues (brain, liver, and kidney) and anisotropic pig tissues (cardiac muscle, tendon, and loin muscle) in transmission and reflection geometries using polarized light imaging. It was elicited that depolarization was greater in anisotropic tissues than in isotropic tissues, demonstrating birefringence-caused depolarization effects.^15^ Mueller algebra was used to quantify the interaction of the polarized light with a tissue sample.^15^ It was affirmed that birefringence, in addition to the various scattering effects, promotes depolarization in anisotropic tissues such as heart muscle and tendon.^15^ Also, Nishizawa et al.^39^ created an endoscope probe based on the spin-light-emitting diode (LED) that uses MC codes to assess scattering events in cancerous and normal biological tissues.

Optical polarimetry of cardiac tissues has been shown to effectively assess the impacts of disease development and monitor treatment efficacy.^11^ Ahmad et al.^16^ quantified factors, such as depolarization and retardance, to characterize myocardial disease such as myocardial infarction and monitored stem cell treatment in radiofrequency ablated (RFA) myocardium. The RFA lesion showed lower depolarization when compared to neighboring healthy tissue.^16^ The reduction in the depolarization was more pronounced at the center of the RFA lesion, which gradually increased toward the healthy tissue at the lesion’s periphery because thermal coagulation mediated by RF ablation leads to tissue anisotropy loss and homogenization of tissue shape.^16^ As a result, incident light polarization is more preserved in RFA lesions, resulting in lower depolarization^16^ due to the underlying morphological characteristics of the tissue.^26^ The myocardium is inherently birefringent because the refractive index along the muscle fibers axis differs from the refractive index in the transverse direction.^40^^,^^41^ Recent works also quantify the linear retardance of infarcted and stem cell therapy treated myocardium.^18^^,^^19^

This paper aims to investigate the use of linearly polarized light for characterizing the bulk optical properties of myocardial tissues in delineating diseased subjects from control. A portable, cost-effective, and rapid linear polarized light-based diagnostic tool is designed and developed. The tool is capable of quantifying the DOLP and accurately distinguishing diseased myocardial tissues from control. An operating wavelength of 850 nm is chosen based on the recent findings,^20^^–^^22^ where 850 nm showed the highest statistical significance in quantifying tissue anisotropy. The developed tool might be utilized in conjunction with endomyocardial biopsy to guide cardiologists to distinguish between healthy and fibrotic heart tissues.

## 2 Methodology and Setup for Experimentation

### 2.1 Preparation of *Ex Vivo* Endomyocardial Biopsy Specimens

The myocardial tissue samples were obtained from healthy (cadaver bodies) and diseased (excised during an endomyocardial biopsy) human subjects with the help of a clinical collaborator from Sri Jayadeva Institute of Cardiovascular Sciences and Research. The diseased tissues were categorized as rheumatic heart disease (RHD) and myxomatous valve (MV) disease, based on preoperative clinical and echocardiographic diagnosis and intraoperative findings. To limit the possibility of infection transmission, the tissue samples were utilized in a controlled environment with a temperature of 25°C and relative humidity of 35% to 40%. Fresh myocardial tissues were kept in a 10% saline solution and iceboxes while being transported from the hospital. The tissue samples were desorbed by placing them on a glass slide for 1 min to remove excess water before performing the measurements. The experiments were carried out in a class 10,000 cleanroom environment.

The average thickness of left ventricle (LV) and right ventricle (RV) tissues were 1.657±0.135 mm and 1.821±0.145  mm for control subjects, 1.396±0.082 mm and 1.493±0.142  mm for RHD subjects, and 0.867±0.142  mm and 0.857±0.098  mm for MV subjects, respectively. Acquiring tissue samples of equal dimensions was extremely challenging, especially due to the limitation posed by the procurement of fresh human heart tissue samples. To mitigate the challenge posed due to varying tissue thickness, a slope-based statistical analysis^42^ was performed to differentiate diseased from control subjects. In this analysis, the DOLP of each sample tissue (control and diseased) was plotted with respect to tissue thickness. A linear fit for the DOLP of each group (control and diseased) was obtained using the fitting approach, which was represented in the form of an equation of a line. Then, the slope was used to distinguish the normal and fibrotic myocardial tissues. This technique could determine the cut-off to distinguish the diseased from control subjects with a certain specificity, sensitivity, and accuracy.^42^

The written patient consensus was obtained through the institutional ethics committees at Sri Jayadeva Institute of Cardiovascular Sciences and Research (SJICR/EC/2021/001) and the Indian Institute of Science (IHEC No: 08/31.03.2021).

### 2.2 Experimental Setup

**Fig. 2:**
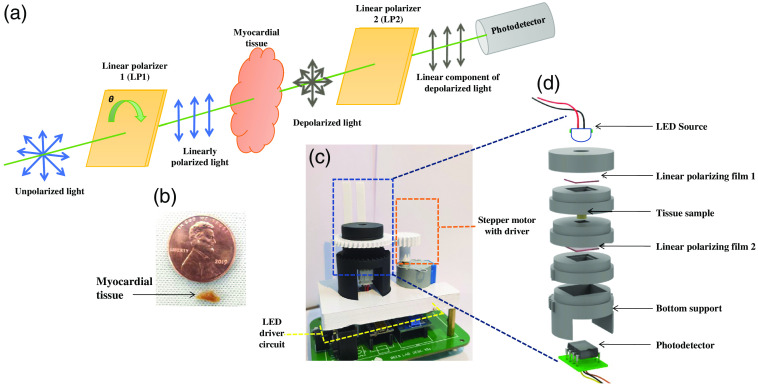
(a) Schematic of the experimental setup of linear polarization-based tool in transmission mode incorporating LP1 and LP2 (linear polarizing films), tissue sample, and photodetector; (b) myocardial tissue; (c) actual photograph; and (d) exploded view of the polarimetric tool.

The experimental setup in the transmission configuration of polarimetric measurements is shown in Fig. 2(a). A narrow beamwidth LED source (SMBB850D-1100-02, Marubeni) emits an unpolarized light that gets converted into a linearly polarized light source using a linear polarizer (LP) film LP1 (LPNIRE2X2, Thor Labs). The LED has a peak operating wavelength of 850 nm, a full-width half-maximum of 37 nm, a radiant intensity of 10 mW, and a viewing half angle of 10 deg, which was utilized as a NIR source. LP1 is placed on a mechanical holder, which was rotated with an interval of 10 deg with the help of a stepper motor unit, as shown in Fig. 2(c). The polarized light source interacts with the heterogeneous heart tissue, whereupon due to the loss of coherence, it yields an unpolarized light. After passing through the second linear polarizer film (LP2), depolarized light emerging from the myocardial tissue becomes linearly polarized.

A silicon photodiode (OPT101 from Texas Instruments) capable of detecting visible and infrared light spectrum (wavelength: 400 to 1100 nm), with a quantum efficiency of 87.5% at 850 nm, is used as a detector.^43^ Figure 2(b) shows the actual picture of heart tissue placed beside a coin, and Fig. 2(c) shows the actual image of the experimental setup that includes the polarimetric tool along with the LED driver circuit and stepper motor unit. Figure 2(d) shows an exploded view of the polarimetric tool.

**Fig. 3:**
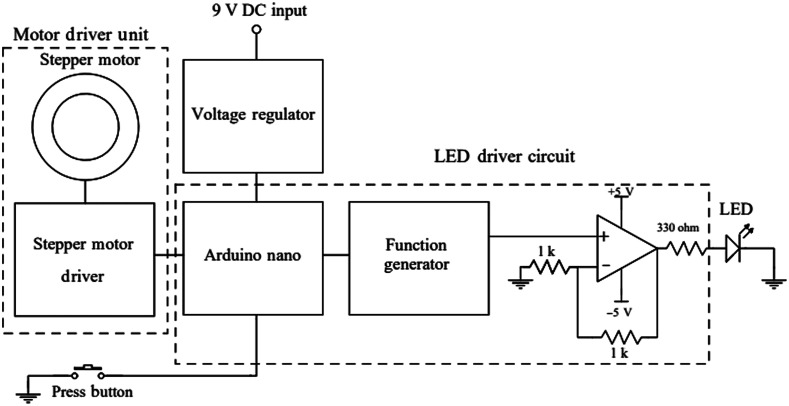
A block schematic of the polarimetric tool and its submodules.

The block diagram for the linear polarization-based tool, along with its constituent modules, is shown in Fig. 3. The stepper motor unit rotates the top platform consisting of the LP1 from 0 deg to 180 deg with an interval of 10 deg using a 3D printed gear assembly. As shown in Fig. 3, the LED driver circuit generates a sinusoidal wave with a frequency of 10 kHz, 2V of peak-to-peak voltage, and 1 V of DC bias to drive the LED. The LED was modulated at 10 kHz to reduce the noise measured due to the ambient light source, hence improving the SNR. To keep the current flowing through the LED at 10 mA, a resistor of 330 Ω was used. The linear voltage regulator (7805) converted the AC supply to a regulated DC supply of 5 V, used to power the function generator (DDS AD9850) along with the Arduino Nano microcontroller. Another linear voltage regulator (7809) gave a controlled 9V DC supply to the silicon photodiode (OPT 101). A 70 MHz, 4-channel digital oscilloscope (GDS-2074E, GW INSTEK) with a sampling rate of 1  GS/s was used to measure the voltage at various angles of rotation. Table 1 gives the specifications of the linear polarization-based tool.

**Table 1:** Specifications of the polarimetric tool.

Requirements	Specifications
Power supply	Mains: 220 V (50 Hz) and +9 V
Dimensions (*L* × *W* × *H*)	100 × 60 × 80 (in mm)
Weight	400 g^a^
Operation type	Continuous wave
Operating wavelength	850 nm
Light source	LED (SMBB850D-1100-02)
Detector	OPT 101
Development Board	Arduino Nano (ATmega-328P microcontroller)
Tissue type	Fresh human heart tissues from the LV and RV
Sensitivity	73.33% for RHD^b^, 91.60% for MV^c^
Specificity	76.92% for RHD^b^, 62.50% for MV^c^
Accuracy	75% for RHD^b^, 80% for MV^c^

a Excluding the weight of digital oscilloscope.

b Rheumatic heart disease.

c Myxomatous valve (MV) disease.

## 3 Theory

Light of any polarization is represented using four Stokes parameters: S1, S2, S3, and S4, where S1 refers to the total intensity of light, whereas S2 conveys the extent of the horizontal linear polarization, S3 refers to 45-deg linear polarization, and S4 represents circular polarization. Stokes vector S is quantified in polarimetry using the flux measurements collected with various polarization analyzers,^44^ which is described as

$$S=[\begin{array}{c} S1\\ S2\\ S3\\ S4 \end{array}]=[\begin{array}{c} I_{\mathrm{HLA}}+I_{\mathrm{VLA}}\\ I_{\mathrm{HLA}}-I_{\mathrm{VLA}}\\ I_{+45\;\mathrm{degLA}}-I_{-45\;\mathrm{degLA}}\\ I_{\mathrm{RCA}}-I_{\mathrm{LCA}} \end{array}],$$ (1)

where $I_{\mathrm{HLA}}$, $I_{\mathrm{VLA}}$, $I_{+45\;\deg\mathrm{LA}}$, $I_{-45\;\deg\mathrm{LA}}$, $I_{\mathrm{RCA}}$, and $I_{\mathrm{LCA}}$ are the light intensities measured in front of the detector with a horizontal linear analyzer ($I_{\mathrm{HLA}}$), vertical linear analyzer ($I_{\mathrm{VLA}}$), a +45  deg-oriented linear analyzer ($I_{+45\;\deg\mathrm{LA}}$), a −45  deg-oriented linear analyzer ($I_{-45\;\deg\mathrm{LA}}$), right circular analyzer ($I_{\mathrm{RCA}}$), and left circular analyzer ($I_{\mathrm{LCA}}$), respectively. Also, $I_{\mathrm{HLA}}+I_{\mathrm{VLA}}=I_{+45\;\deg\mathrm{LA}}+I_{-45\;\deg\mathrm{LA}}=I_{\mathrm{RCA}}+I_{\mathrm{LCA}}=S1$, where S1 is the light intensity measured without an analyzer in front of the detector.

The DOLP is defined as the measure of light that still retains its original polarization state after interacting with a turbid medium,^44^ such as myocardial tissues used in this study. The DOLP can be evaluated from the Stokes vector (S) as

$$\mathrm{DOLP}=\frac{\sqrt{S2^{2}+S3^{2}}}{S1},$$ (2)

where S1, S2, and S3 are Stokes parameters defined in Eq. (1). DOLP should be between 0 and 1, with zero representing unpolarized light and 1 being fully polarized light.

## 4 Results and Discussions

The experiments were performed on fresh heart tissue samples from N=17 subjects, where N=7 were control subjects, N=7 were subjects suffering from RHD, and N=3 were subjects suffering from MV disease.

**Fig. 4:**
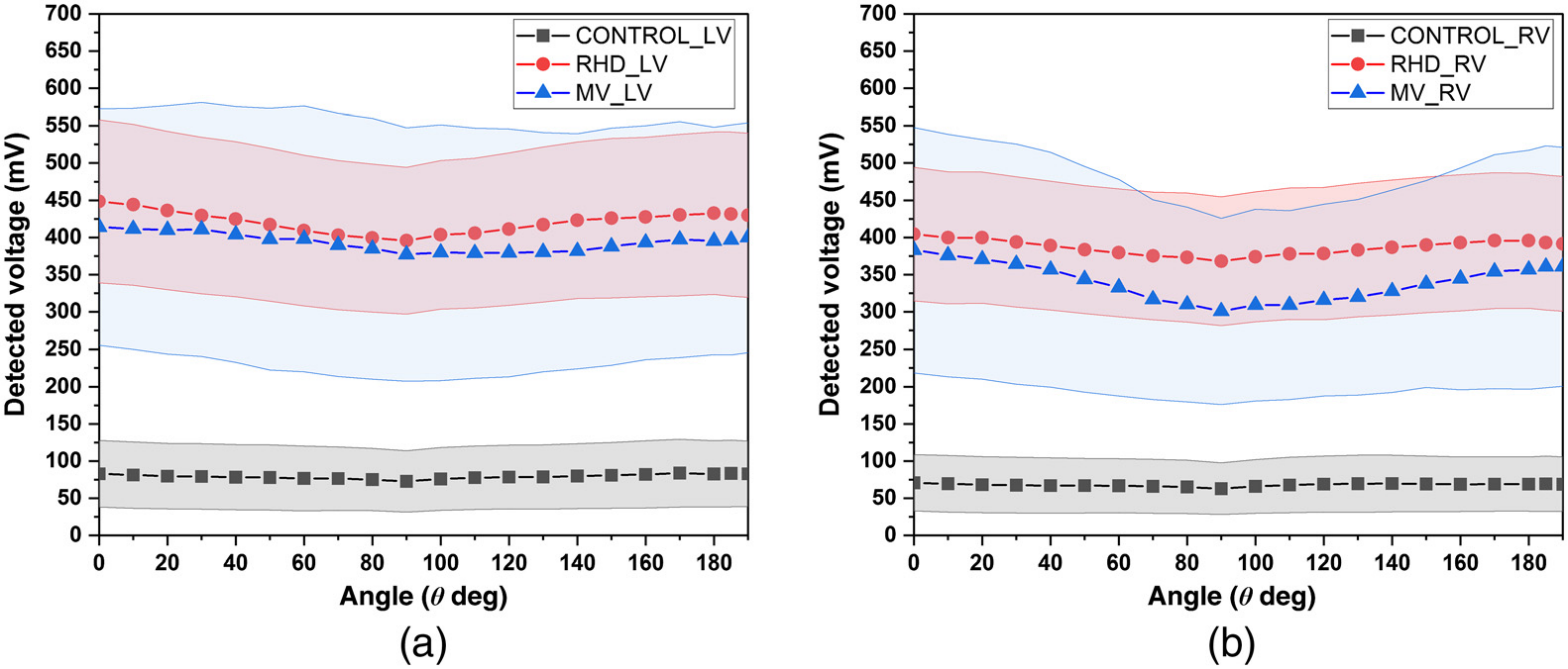
Detected voltages with error band for (a) LV tissue samples of control, RHD, and MV subjects and (b) RV tissue samples of control, RHD, and MV subjects.

Figures 4(a) and 4(b) show the average detected voltages (in mV) as a function of rotation (θ) for LV and RV tissue samples of control, RHD, and MV subjects. The measured voltages differed significantly between control and diseased subjects (RHD and MV).

The DOLP for control, RHD, and MV subjects were calculated using Eq. (2) and tabulated in Tables 2–4, respectively.

**Table 2:** DOLP for LV and RV tissue samples of control subjects.

Control subjects	DOLP for LV	DOLP for RV
C1	0.135	0.075
C2	0.11	0.078
C3	0.076	0.076
C4	0.091	0.064
C5	0.066	0.029
C6	0.093	0.066
C7	0.046	0.030

**Table 3:** DOLP for LV and RV tissue samples of subjects with RHD.

Subjects	DOLP for LV	DOLP for RV
D1	0.195	0.179
D2	0.102	0.069
D3	0.032	0.052
D4	0.1	0.044
D5	0.026	0.03
D6	0.069	0.073
D7	0.103	0.093

**Table 4:** DOLP for LV and RV tissue samples of subjects with MV.

Subjects	DOLP for LV	DOLP for RV
M1	0.218	0.157
M2	0.039	0.066
M3	0.035	0.14

**Fig. 5:**
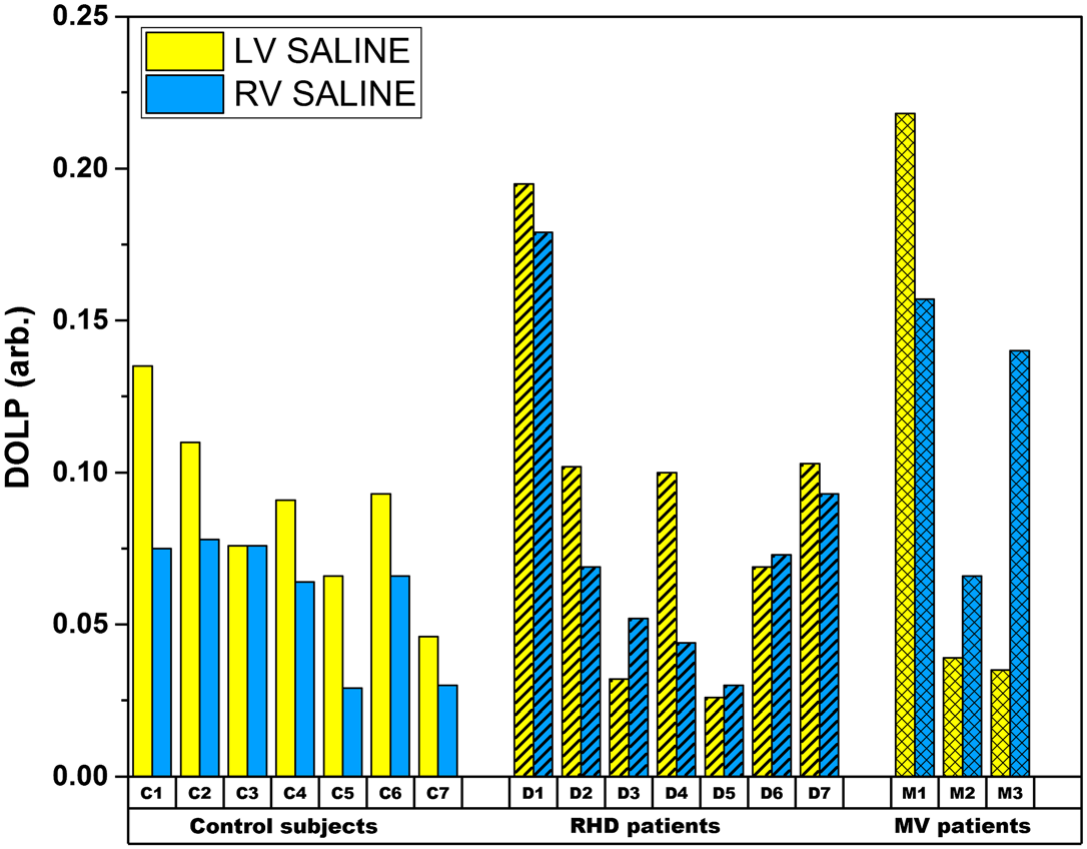
Comparison plot of DOLP for LV and RV tissue samples of control, RHD, and MV subjects.

Figure 5 summarizes the comparison of the absolute magnitudes of DOLP between the LV and RV for the seven control subjects (C1–C7), seven RHD subjects (D1–D7), and three MV subjects (M1–M3). However, to mitigate the effect of tissue thickness, the DOLP for the control subjects with RHD and MV diseased subjects are discussed in the following sections:

### 4.1 Control subjects versus Diseased Group 1 (RHD Subjects)

The average DOLP of the control subjects (N=7) are evaluated as 0.088±0.011 and 0.059±0.008 for LV and RV tissues, respectively. The average DOLP of the LV tissues of RHD (N=7) subjects (0.089±0.022) is observed to be similar to that of control subjects. For the RV tissues, however, RHD subjects have a greater average DOLP (0.077±0.019) than the control subjects. One of the reasons for the lower DOLP in the control subjects may be due to the well-aligned arrangement of cardiomyocytes surrounded by ECM components in the healthy heart tissue.^11^ However, disruption of this well-aligned arrangement, loss in cardiomyocytes, and an increase in collagen production due to fibrosis^16^ results in lesser depolarization, which may have contributed to the greater average DOLP of RV tissues in RHD subjects.

When compared to the non-rheumatic control subjects, rheumatic subjects’ mitral valves (in RHD subjects) had a greater deposition of collagen (type I and type III), indicating fibrosis.^45^ TGF-β, one of several cytokines implicated in the inflammatory process in RHD, is related to valvular fibrosis in promoting the activation of myofibroblasts and the formation of collagen.^46^

**Fig. 6:**
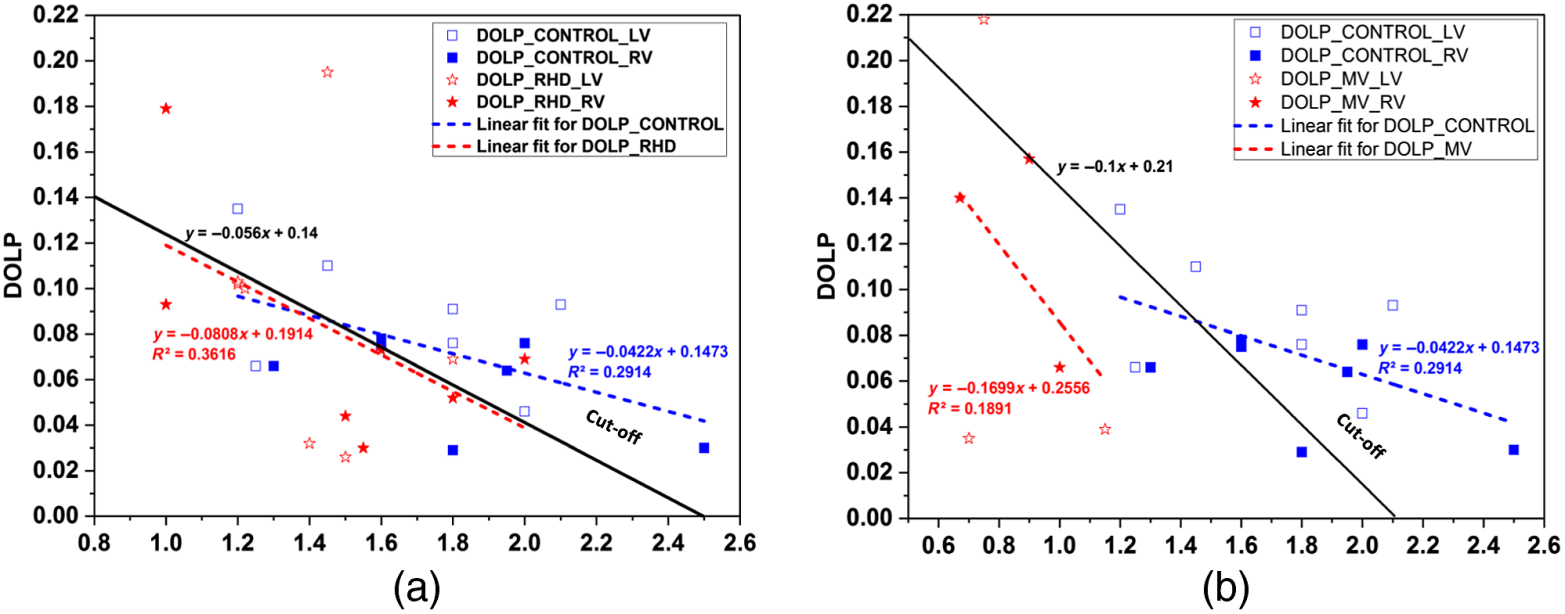
Plot for DOLP values versus thickness of LV and RV tissue samples for (a) control subjects and RHD subjects and (b) control subjects and MV subjects.

Figure 6(a) shows the variation of DOLP with the sample tissue thickness in control and RHD subjects. The blue and red dashed lines in Fig. 6(a) showcase the linear fit for the control and RHD subjects, respectively. The slope of the linear fit for control subjects ($-0.0422\;{\mathrm{mm}}^{-1}$) is approximately half of that for the RHD subjects ($-0.0808\;{\mathrm{mm}}^{-1}$). The black line with a slope ($-0.056\;{\mathrm{mm}}^{-1}$) between the control and RHD is used to distinguish between the control and RHD groups. The slope of the black line is used as a cut-off to distinguish between the control and RHD, a basis to quantify the sensitivity, specificity, and accuracy. The sensitivity, specificity, and accuracy in delineating the control group from the RHD group are found to be 73.33%, 76.92%, and 75%, respectively.

### 4.2 Control Subjects Versus Diseased Group 2 (MV Subjects)

The average DOLP values for LV and RV tissues of MV subjects (N=3) are 0.097±0.060 and 0.121±0.028, respectively. The average DOLP for the MV subjects is higher than the average DOLP of control subjects (0.088±0.011 and 0.059±0.008 for LV and RV tissues, respectively). A higher DOLP for diseased tissues than healthy tissues is consistent with the prior findings,^47^ which reveal a reduction in tissue anisotropy as healthy heart muscle is replaced with infarcted cardiac tissue. This occurs as a result of a significant disruption in the tissue architecture.^47^ In subjects with MV, lesions that produce regurgitation in the mitral valve are caused by gradual thickening generated by activated myofibroblasts.^48^ This results in myxomatous alterations. In the diseased myocardial tissues, the loss of cardiomyocytes and increased collagen buildup in the ECM result in lower scattering of the incident polarized light, leading to lower depolarization and higher DOLP.

Figure 6(b) shows the variation of DOLP with the sample tissue thickness in control and MV subjects. The blue and red dashed lines in Fig. 6(b) showcase the linear fit for the control and MV subjects, respectively. The slope of the linear fit for control subjects ($-0.0422\;{\mathrm{mm}}^{-1}$) is found to be approximately one-fourth of that for the MV subjects ($-0.1699\;{\mathrm{mm}}^{-1}$). The black line with a slope ($-0.1\;{\mathrm{mm}}^{-1}$) that lies between the slope of the control and MV is used as a cut-off to distinguish between the control and MV subjects. The sensitivity, specificity, and accuracy in delineating the control group from the MV group are 91.6%, 62.5%, and 80%, respectively.

## 5 Conclusions

Optical polarimetry of cardiac tissues evaluates the anisotropy within the myocardium to quantify the extent of fibrosis. Adopting such optical polarimetry-based tools provides the cardiologists with supplementary insights to make an informed assessment. A robust, cost-effective, and simple-to-use diagnostic tool is designed and developed to delineate the diseased groups (RHD and MV disease) from the control group with a high statistical significance. The tool is capable of distinguishing the diseased and control group by quantifying the DOLP of myocardial tissues. The measurements are carried out on tissue samples from the LV and RV of N=7 control subjects and diseased subjects (N=7: RHD, and N=3: MV). The sensitivity, specificity, and accuracy in delineating the control group from the RHD group are found to be 73.33%, 76.92%, and 75%, respectively, and from the MV group are 91.6%, 62.5%, and 80%, respectively. This study needs to be followed up with the measurements on a large cohort of healthy and diseased subjects. We envisage the design and development of a probe that can quantify the DOLP of the *in vivo* heart tissue during the surgery.

## Code, Data, and Materials Availability

Raw data can be obtained upon reasonable request.

## References

[1] Roth G. A. et al., “Global burden of cardiovascular diseases and risk factors, 1990–2019,” J. Am. Coll. Cardiol. 76, 2982–3021 (2020). JACCDI 0735-1097 10.1016/j.jacc.2020.11.010 33309175 PMC7755038

[2] Karamitsos T. D. et al., “Myocardial tissue characterization and fibrosis by imaging,” JACC Cardiovasc. Imaging 13, 1221–1234 (2020). 10.1016/j.jcmg.2019.06.030 31542534

[3] Murtha L. A. et al., “The processes and mechanisms of cardiac and pulmonary fibrosis,” Front. Physiol. 8, 777 (2017). FROPBK 0301-536X 10.3389/fphys.2017.00777 29075197 PMC5643461

[4] Hinderer S. Schenke-Layland K., “Cardiac fibrosis—a short review of causes and therapeutic strategies,” Adv. Drug Deliv. Rev. 146, 77–82 (2019). ADDREP 0169-409X 10.1016/j.addr.2019.05.011 31158407

[5] Hein S. Schaper J., “The extracellular matrix in normal and diseased myocardium,” J. Nucl. Cardiol. 8, 188–196 (2001). JNCAE2 1071-3581 10.1067/mnc.2001.113331 11295697

[6] Cohn J. N. Ferrari R. Sharpe N., “Cardiac remodeling—concepts and clinical implications: a consensus paper from an international forum on cardiac remodeling. Behalf of an International Forum on Cardiac Remodeling,” J. Am. Coll. Cardiol. 35, 569–582 (2000). JACCDI 0735-1097 10.1016/S0735-1097(99)00630-0 10716457

[7] Rockey D. C. Bell P. D. Hill J. A., “Fibrosis—a common pathway to organ injury and failure,” N. Engl. J. Med. 372, 1138–1149 (2015). NEJMAG 0028-4793 10.1056/NEJMra1300575 25785971

[8] Pattanayak P. Bleumke D. A., “Tissue characterization of the myocardium,” Radiol. Clin. North Am. 53, 413–423 (2015). 10.1016/j.rcl.2014.11.005 25727003 PMC4348002

[9] Graham-Brown M. P. M. et al., “Imaging of myocardial fibrosis in patients with end-stage renal disease: current limitations and future possibilities,” BioMed. Res. Int. 2017, 5453606 (2017). 10.1155/2017/5453606 28349062 PMC5352874

[10] Sankaran V. Walsh J. T. Maitland D. J., “Comparative study of polarized light propagation in biologic tissues,” J. Biomed. Opt. 7(3), 300–306 (2002). JBOPFO 1083-3668 10.1117/1.1483318 12175278

[11] Ahmad I., “Review of the emerging role of optical polarimetry in characterization of pathological myocardium,” J. Biomed. Opt. 22, 100901 (2017). JBOPFO 1083-3668 10.1117/1.JBO.22.10.100901 29076304

[12] Bickel W. S. et al., “Application of polarization effects in light scattering: a new biophysical tool,” Proc. Natl. Acad. Sci. U. S. A. 73, 486–490 (1976). 10.1073/pnas.73.2.486 813228 PMC335934

[13] Fleming C. P. et al., “*In vitro* characterization of cardiac radiofrequency ablation lesions using optical coherence tomography,” Opt. Express 18, 3079 (2010). OPEXFF 1094-4087 10.1364/OE.18.003079 20174138

[r14] Fleming C. P. Quan K. J. Rollins A. M., “Toward guidance of epicardial cardiac radiofrequency ablation therapy using optical coherence tomography,” J. Biomed. Opt. 15, 041510 (2010). JBOPFO 1083-3668 10.1117/1.3449569 20799788 PMC2912935

[r15] Alali S., “Quantitative correlation between light depolarization and transport albedo of various porcine tissues,” J. Biomed. Opt. 17, 045004 (2012). JBOPFO 1083-3668 10.1117/1.JBO.17.4.045004 22559678

[16] Ahmad I. et al., “Polarimetric assessment of healthy and radiofrequency ablated porcine myocardial tissue,” J. Biophotonics 9, 750–759 (2016). 10.1002/jbio.201500184 26394151

[17] Sanchez-Cano A. et al., “Measurement method of optical properties of *ex vivo* biological tissues of rats in the near-infrared range,” Appl. Opt. 59, D111–D117 (2020). APOPAI 0003-6935 10.1364/AO.384614 32400631

[18] Ghosh N. et al., “Mueller matrix decomposition for polarized light assessment of biological tissues,” J. Biophotonics 2, 145–156 (2009). 10.1002/jbio.200810040 19343695

[19] Wood M. F. G. et al., “Polarization birefringence measurements for characterizing the myocardium, including healthy, infarcted, and stem-cell-regenerated tissues,” J. Biomed. Opt. 15, 047009 (2010). JBOPFO 1083-3668 10.1117/1.3469844 20799840

[20] Pal U. M. et al., “Hybrid spectral-IRDx: near-IR and ultrasound attenuation system for differentiating breast cancer from adjacent normal tissue,” IEEE Trans. Biomed. Eng. 68, 3554–3563 (2021). IEBEAX 0018-9294 10.1109/TBME.2021.3077582 33945469

[r21] Kamal A. M. et al., “Towards development of LED-Based time-domain near-IR spectroscopy system for delineating breast cancer from adjacent normal tissue,” IEEE Sens. J. 21, 17758–17765 (2021). ISJEAZ 1530-437X 10.1109/JSEN.2021.3082850

[22] Kamal A. M. et al., “Toward the development of portable light emitting diode‐based polarization spectroscopy tools for breast cancer diagnosis,” J. Biophotonics 15, e202100282 (2022). 10.1002/jbio.202100282 34846777

[23] Sprenger J. et al., “Toward a quantitative method for estimating tumour-stroma ratio in breast cancer using polarized light microscopy,” Biomed. Opt. Express 12, 3241–3252 (2021). BOEICL 2156-7085 10.1364/BOE.422452 34221657 PMC8221948

[r24] Westreich J. et al., “Novel methodology to image stromal tissue and assess its morphological features with polarized light: towards a tumour microenvironment prognostic signature,” Biomed. Opt. Express 10, 3963–3973 (2019). BOEICL 2156-7085 10.1364/BOE.10.003963 31452988 PMC6701544

[25] Jones B. et al., “Novel quantitative signature of tumor stromal architecture: polarized light imaging differentiates between myxoid and sclerotic human breast cancer stroma,” Biomed. Opt. Express 11, 3246–3262 (2020). BOEICL 2156-7085 10.1364/BOE.392722 32637252 PMC7316019

[26] Sharma M. et al., “Histopathological correlations of bulk tissue polarimetric images: case study,” J. Biophotonics 14, e202000475 (2021). 10.1002/jbio.202000475 33533565

[27] Demos S. G. Sharareh S., “Real time assessment of RF cardiac tissue ablation with optical spectroscopy,” Opt. Express 16, 15286 (2008). OPEXFF 1094-4087 10.1364/OE.16.015286 18795066

[28] John P. et al., “Glucose sensing in *ex-vivo* human gingival tissue with enhanced sensitivity in combination band,” IEEE Sens. J. 19, 7347–7354 (2019). ISJEAZ 1530-437X 10.1109/JSEN.2019.2917378

[29] Ivanov D. et al., “Polarization and depolarization metrics as optical markers in support to histopathology of *ex vivo* colon tissue,” Biomed. Opt. Express 12, 4560 (2021). BOEICL 2156-7085 10.1364/BOE.426713 34457432 PMC8367259

[30] Li P. et al., “Analysis of tissue microstructure with Mueller microscopy: logarithmic decomposition and Monte Carlo modeling,” J. Biomed. Opt. 25, 015002 (2020). JBOPFO 1083-3668 10.1117/1.JBO.25.1.015002 PMC7008502 31933331

[31] Borovkova M. et al., “Evaluating β-amyloidosis progression in Alzheimer’s disease with Mueller polarimetry,” Biomed. Opt. Express 11, 4509 (2020). BOEICL 2156-7085 10.1364/BOE.396294 32923060 PMC7449745

[32] Lee H. R. et al., “Mueller matrix imaging for collagen scoring in mice model of pregnancy,” Sci. Rep. 11, 15621 (2021). SRCEC3 2045-2322 10.1038/s41598-021-95020-8 34341418 PMC8329204

[33] Ivanov D. et al., “Colon cancer detection by using Poincaré sphere and 2D polarimetric mapping of *ex vivo* colon samples,” J. Biophotonics 13, e202000082 (2020). 10.1002/jbio.202000082 32390327

[34] Ushenko V. A. et al., “Embossed topographic depolarisation maps of biological tissues with different morphological structures,” Sci. Rep. 11, 3871 (2021). SRCEC3 2045-2322 10.1038/s41598-021-83017-2 33594107 PMC7886906

[35] Roa C. et al., “Auto-detection of cervical collagen and elastin in Mueller matrix polarimetry microscopic images using K-NN and semantic segmentation classification,” Biomed. Opt. Express 12, 2236 (2021). BOEICL 2156-7085 10.1364/BOE.420079 33996226 PMC8086465

[36] Gonzalez M. et al., “Design and implementation of a portable colposcope Mueller matrix polarimeter,” J. Biomed. Opt. 25, 116006 (2020). JBOPFO 1083-3668 10.1117/1.JBO.25.11.116006 PMC7666868 33191686

[37] Panigrahi S. Gioux S., “Machine learning approach for rapid and accurate estimation of optical properties using spatial frequency domain imaging,” J. Biomed. Opt. 24, 071606 (2018). JBOPFO 1083-3668 10.1117/1.JBO.24.7.071606 PMC6995874 30550050

[38] Nguyen M. H. et al., “Machine learning to extract physiological parameters from multispectral diffuse reflectance spectroscopy,” J. Biomed. Opt. 26, 052912 (2021). JBOPFO 1083-3668 10.1117/1.JBO.26.5.052912

[39] Nishizawa N. et al., “Monte Carlo simulation of scattered circularly polarized light in biological tissues for detection technique of abnormal tissues using spin-polarized light emitting diodes,” Jpn. J. Appl. Phys. 59, SEEG03 (2020). 10.35848/1347-4065/ab69db

[40] Bosman S., “Heat-induced structural alterations in myocardium in relation to changing optical properties,” Appl. Opt. 32, 461 (1993). APOPAI 0003-6935 10.1364/AO.32.000461 20802712

[41] Haskell R. C. Carlson F. D. Blank P. S., “Form birefringence of muscle,” Biophys. J. 56, 401–413 (1989). BIOJAU 0006-3495 10.1016/S0006-3495(89)82686-4 2775834 PMC1280489

[42] Zhou X.-H. Obuchowski N. A. McClish D. K., Statistical Methods in Diagnostic Medicine, John Wiley & Sons, Inc., Hoboken, New Jersey (2014).

[43] Li T. et al., “A brief review of OPT101 sensor application in near-infrared spectroscopy instrumentation for intensive care unit clinics,” Sensors 17, 1701 (2017). SNSRES 0746-9462 10.3390/s17081701 PMC5580117 28757564

[44] Tuchin V. V., “Polarized light interaction with tissues,” J. Biomed. Opt. 21, 071114 (2016). JBOPFO 1083-3668 10.1117/1.JBO.21.7.071114 27121763

[45] Banerjee T. et al., “Clinical significance of markers of collagen metabolism in rheumatic mitral valve disease,” PLoS One 9, e90527 (2014). POLNCL 1932-6203 10.1371/journal.pone.0090527 24603967 PMC3948343

[46] Passos L. S. A. Nunes M. C. P. Aikawa E., “Rheumatic heart valve disease pathophysiology and underlying mechanisms,” Front. Cardiovasc. Med. 7, 612716 (2021). 10.3389/fcvm.2020.612716 33537348 PMC7848031

[47] Wallenburg M. A. et al., “Two-photon microscopy of healthy, infarcted and stem-cell treated regenerating heart,” J. Biophotonics 4, 297–304 (2011). 10.1002/jbio.201000059 20827682

[48] Rajamannan N. M., “Myxomatous mitral valve disease bench to bedside: LDL-density-pressure regulates Lrp5,” Expert Rev. Cardiovasc. Ther. 12, 383–392 (2014). 10.1586/14779072.2014.893191 24575776 PMC4048944

